# Life Table and Preference Choice of *Frankliniella occidentalis* (Thysanoptera: Thripidae) for Kidney Bean Plants Treated by Exogenous Calcium

**DOI:** 10.3390/insects12090838

**Published:** 2021-09-17

**Authors:** Guang Zeng, Jun-Rui Zhi, Mao Ye, Wen Xie, Tao Zhang, Ding-Ying Li, Li Liu, Xiao-Bao Wu, Yu Cao

**Affiliations:** 1 The Provincial Key Laboratory for Agricultural Pest Management of the Mountainous Region, Institute of Entomology, Guizhou University, Guiyang 550005, China; zengguang1992@126.com (G.Z.); xiewen694226217@163.com (W.X.); zhangtao3185@126.com (T.Z.); lidingyin553100@163.com (D.-Y.L.); liuli92@outlook.com (L.L.); XiaobaoWU1995@163.com (X.-B.W.); 2 Guizhou Provincial Key Laboratory for Rare Animal and Economic Insect of the Mountainous Region, Department of Biology and Engineering of Environment, Guiyang University, Guiyang 550005, China; yucaosuccess@126.com

**Keywords:** *Frankliniella occidentalis*, exogenous calcium, inducible plant defense, life table, preference choice

## Abstract

**Simple Summary:**

Western flower thrips, *Frankliniella occidentalis*, is an invasive key pest that damages vegetables and ornamentals worldwide. The activation of induced resistance by chemicals may provide a simple and feasible way of achieving improvement of resistance to stress in crop plants, which is an important technology for the development of sustainable agriculture. Calcium (Ca) is an essential element for plants; numerous studies have shown that Ca can confer crop plants with resistance to abiotic and biotic stresses. For the first time, we report the negative effects of exogenous Ca on kidney bean plants in relation to the performance of *F. occidentalis*, including a reduced preference of thrips. Therefore, Ca could potentially be used to control *F. occidentalis*.

**Abstract:**

Exogenous calcium (Ca) has been used to induce host plant resistance in response to abiotic and biotic stresses, including from thrips attack. The aim of this study was to determine whether exogenously applied Ca affects the performance of *Frankliniella occidentalis*. We assessed the development time, total longevity, reproduction, and population parameters of *F. occidentalis*, and its preference choice on Ca-treated or untreated control kidney bean plants under laboratory conditions. The results showed that *F. occidentalis* fed on Ca-treated leaves had a longer developmental time but lower longevity (female and male) and fecundity than *F. occidentalis* fed on control leaves. Population parameters, including the intrinsic rate of increase (*r*), finite rate of increase (*λ*), and net reproductive rate (*R*_0_), were all found higher in control leaves than in Ca-treated leaves, and the mean generation time (*T*) was shorter. In preference choices, the number of thrips on control plants was higher than the number of thrips on Ca-treated kidney bean plants. Overall, our results indicated that exogenous Ca pretreatment on kidney bean plants affected the life history and preference choice of *F. occidentalis*, suggesting Ca might be used as a promising elicitor of inducible plant defense against thrips.

## 1. Introduction

*Frankliniella occidentalis* (Pergande) (Thysanoptera: Thripidae) is a significant agricultural invasive pest worldwide due to its broad host range and rapid reproduction, and it poses a great threat to crops [1,2]. Thrips feeding results in damaged areas with a silvery appearance because of thrips piercing and sucking plant leaves and the increased possibility of destructive disease that causes serious yield losses [3,4]. In China, *F. occidentalis* has quickly spread to more than 10 provinces in the last 10 years since its initial occurrence in Beijing in 2003 and has been found in the previously assumed to be unsuitable regions of northwest and northeast of China [5]. Chemical insecticides have predominantly been used to control *F. occidentalis* as the primary control measure [6,7]. Currently, *F. occidentalis* is difficult to control due to its rapid development of resistance to many insecticides and its hidden nature of feeding [2,6]. Relying on plant defenses against *F. occidentalis* has been discussed as a promising alternative for thrips control because plant defenses provide a sustainable control method for the future [8,9].

Enhancement of constitutive or inducible host plant defenses against *F. occidentalis* may be critical for sustainable thrips control [8,10]. Improving plant resistance by inducing defense mechanisms allows plants to manage energy reserves only when and as needed, and it has been actively studied in many crops [8,11]. Numerous studies have shown that the application of exogenous abiotic elicitors can induce plant resistance to insect herbivores [9]. For instance, exogenously applied silicon improved the resistance of rice plants to yellow stem borer *Scirpophaga incertulas* (Lepidoptera: Crambidae) and caterpillar *Cnaphalocrocis medinalis* (Lepidoptera: Pyralidae) by increasing larval mortality and reducing larval mass, respectively [12,13]. Both longer development time and lower numbers of aphids *Diuraphis noxia* (Homoptera: Aphididae) were observed on wheat plants treated with exogenous potassium, in comparison with untreated plants [14]. In the field, both exogenously applied silicon and potassium enhanced the plant resistance of wheat and significantly decreased the *Sesamia inferens* (Lepidoptera: Noctuidae) incidence with a lower percent white ear damage [15]. The application of biochar also led to lower levels of reproduction of the aphid *Sitobion avenae* (Homoptera: Aphididae) on wheat [16].

Calcium (Ca) is an essential substance for plant growth and has been shown to improve plant tolerance to many abiotic and biotic stresses. The application of exogenous Ca could improve plant resistance against abiotic stresses, such as low temperature, high temperature, salt stress, heavy metal damage, and herbicide damage [17,18,19,20]. The application of exogenous Ca could also regulate plant resistance against biotic stress. For example, fewer and smaller lesions were observed in wheat leaves pretreated with nutrient solution containing Ca [21]. Ca pretreatment remarkably decreased the growth of the bacterial wilt and powder mildew in tomato plants [22,23]. Moreover, exogenous Ca application in sugarcane reduced the borer mass of *Eldana saccharina* (Lepidoptera: Pyralidae) and stalk damage [24]. Likewise, Ca pretreatment enhanced plant response against wheat aphid *Schizaphis graminum* (Hemiptera: Aphididae) attack by modulating callose deposition in the phloem [25]. Our previous study showed that the application of Ca also increased the tolerance of kidney bean plants to thrips and promoted the synthesis of defense compounds [26]. However, Ca treatment on kidney bean plants and the potential effect on the performance of *F. occidentalis* remain unclear. To date, most of the literature focuses on the effects of temperature, host plants, or natural enemies on life table studies for herbivores. Less is known on the impact on the parameters examined in life tables of a herbivore from induced host plant defense by an exogenous elicitor. Therefore, our objectives were to investigate the life table parameters of *F. occidentalis* on kidney bean leaf exposed to exogenous Ca. Additionally, the preference of *F. occidentalis* was determined through choice tests between the control plant and the Ca-treated kidney bean plant. Additional efforts are required to explore more effective and environmentally friendly methods to control thrips.

## 2. Materials and Methods

### 2.1. Insect Rearing

*Frankliniella occidentalis* was originally collected from vegetable plants in the Guiyang area of Guizhou Province, China, maintained on kidney bean pods, and kept in a climatic chamber at a constant temperature of 25 ± 1 °C, 14 h: 10 h light–dark photoperiod and 70% relative humidity. The populations were continuously reared for more than 20 generations in the laboratory. Adult females and males of *F. occidentalis* were used as sources for the bioassays.

### 2.2. Plant

Seeds of kidney bean (*Phaseolus vulgaris* L.) were presoaked in sterile distilled water for 1 d and pregerminated for 2 d at 30 °C, and then were transplanted individually into separate plastic pots (12 cm diameter, 15 cm height) filled with nutrient soil in the nursery of the Institute of Entomology, Guizhou University, China, at 25 °C and 14 h: 10 h light–dark photoperiod. Commercially obtained nutrient soil was used and autoclaved at 121 °C before use. All plants were watered daily. No pesticides were applied during cultivation, and kidney bean plants with two leaves (15 d) and four leaves (30 d) were used for the growth and development and choice tests of thrips, respectively.

### 2.3. Life Table Study

#### 2.3.1. Thrips Development Duration and Nymphal Survival

Kidney bean plants at the two-leaf stage were presprayed with 10 mL H_2_O (untreated control) or 10 mL of 20 mM CaCl_2_ solution. After 24 h, each leaf was detached and placed in a separate Petri dish (9 cm diameter, 2 cm height). The petiole of each leaf was wrapped using moist cotton. Then, each leaf was infested by one female and one male of *F. occidentalis* (3-day-old adult) selected randomly from the thrips culture, and the Petri dish was immediately sealed with a parafilm membrane to prevent the thrips from escaping. The adults were removed at 12 h after treatment. These leaves were observed every 12 h to determine the hatching of the eggs of thrips. The developmental period of the eggs was recorded by determining the passage of time until the appearance of the first nymphs [27]. After egg hatching, one neonate was retained on each leaf to observe nymphal development duration and survival rates. In total, 60 new nymphs were used on leaves with Ca treatment plus an additional 60 nymphs on untreated leaves as a control. The developmental time of nymphs in each treatment was observed every 12 h until they developed into the adult stage as described by Zeng et al. [26]. Thrips immature stages were observed using a stereoscope microscope. All rearing was maintained at 25 ± 1 °C, a 14 h:10 h light–dark photoperiod, and 70% relative humidity.

#### 2.3.2. Thrips Longevity and Fecundity

At adult emergence, one male and one female from the same treatment were randomly collected and paired and placed in a new Petri dish containing pretreated and control leaves. Those leaves were changed and replaced every day. Each leaf having newly deposited *F. occidentalis* eggs was carefully collected and individually transferred to new Petri dishes containing moist filter paper, and the Petri dishes were sealed with parafilm. The survival of females and males was checked and recorded every day until they died. The number of eggs that hatched daily by each female were also counted to estimate the numbers of offspring.

### 2.4. Choice Test

In this experiment, four-leaf plants were used and individually sprayed with 20 mL of 20 mM CaCl_2_ solution. Control plants were sprayed with 20 mL of H_2_O. After 1 d, one Ca-treated plant and one untreated plant were placed into nylon-gauze cages (200 mesh, 50 cm length × 50 cm width × 50 cm height), and 50 adult female thrips (3-day-old adults) were immediately placed halfway between a control plant and a Ca-treated plant. After 24 h, the number of adults that had settled on Ca-treated plants and control plants was observed and recorded. The experiment had 12 replicates and was performed in a greenhouse at 25 °C with a 14 h:10 h light–dark photoperiod.

### 2.5. Data Analysis

#### 2.5.1. Life Table Analyses

The development time, longevity, fecundity, and life table parameters of *F. occidentalis* were analyzed based on the age-stage, two-sex life table theory [28,29]. The details of the population parameter calculation, definitions, and equations are presented in Ding et al. [30]. The population parameters including the intrinsic rate of increase (*r*), finite rate of increase (*λ*), net reproductive rate (*R*_0_), mean generation time (*T*), age-stage-specific survival rate (*s_xj_*, where *x* = age and *j* = stage), age-specific survival rate (*l_x_*), age-stage-specific fecundity (*f_xj_*), age-specific fecundity (*m_x_*), age-specific net maternity (*l_x_m_x_*), age-stage-specific life expectancy (*e_xj_*), and age-stage-specific reproductive value (*v_xj_*) were calculated using raw daily life history data [28,31,32]. The variances and standard errors of population parameters were estimated using a bootstrap method with 100,000 resamplings [31]. Differences between Ca-treated leaves and control leaves were calculated by using the paired bootstrap test based on the 5% significance level [32,33]. All of these analyses were performed by TWOSEX-MSChart [34].

#### 2.5.2. Choice Tests

For choice bioassays, the number of thrips on Ca-treated plants and control plants was subjected to a *χ*^2^ test (** *p* < 0.01, * *p* < 0.05) and was evaluated using SPSS version 22.0 (SPSS Inc., Chicago, IL, USA).

## 3. Results

### 3.1. Developmental and Reproductive Parameters

#### 3.1.1. Development Duration

There were differences in thrips developmental time between the two treatments (Table 1). The developmental duration of eggs, second instar, and immature (all immature stages combined) stages of thrips fed on Ca-treated leaves were significantly longer than thrips fed on control leaves but not for first instar, prepupa, and pupa stages. No significant differences were observed for the survival rates from egg to adult (preadult survival rate) (Table 1). The longevity of females and males fed Ca-treated leaves was significantly shorter than the longevity of females and males fed control leaves (Table 1). There were significant differences in the total preoviposition period (TPOP) between Ca-treated and control leaves but not the adult preoviposition period (APOP) (Table 1). The mean fecundity of females exposed to control leaves was 80.29 eggs, which was significantly higher than the mean fecundity of females exposed to Ca-treated leaves (55.34 eggs), and fewer oviposition days were observed when the thrips fed on Ca-treated leaves than on control leaves (Table 1).

#### 3.1.2. Age-Specific Survivorship, Life Expectancy, and Fecundity

**Figure 1:**
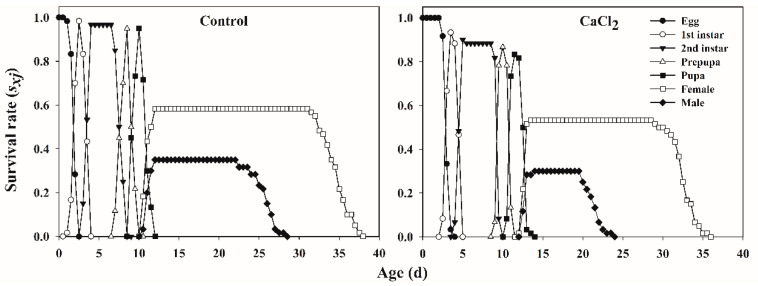
The age-stage-specific survival rate (*s_xj_*) of *Frankliniella occidentalis* fed on control and Ca-treated kidney bean leaves.

The age-stage-specific survival rate (*s_xj_*) of *F. occidentalis* fed on Ca-treated leaves and control leaves showed the probability that a newly laid egg would survive to age *x* and stage *j* (Figure 1), and the curves showed obvious overlaps between successive life stages due to the variable developmental rates among different thrips individuals. The *s_x_j* of females and males was similar in Ca-treated leaves and control leaves, and the survival rate of adults fed Ca-treated leaves was lower than the survival rate of adults fed control leaves (Figure 1).

**Figure 2:**
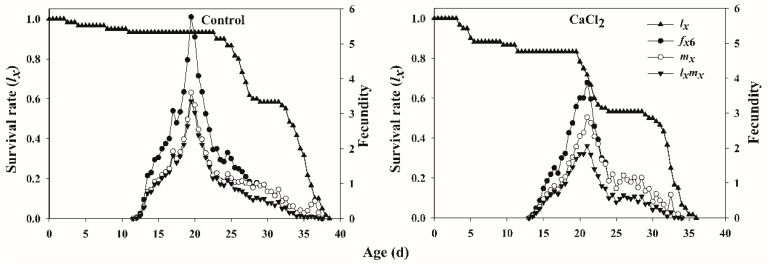
The age-specific survival rate (*l_x_*), age-stage-specific fecundity (*f_xj_*), age-specific fecundity (*m_x_*), and age-specific net maternity (*l_x_m_x_*) of *Frankliniella occidentalis* fed on control and Ca-treated kidney bean leaves.

The age-specific survival rate (*l_x_*), age-stage-specific fecundity (*f_x_*_6_), age-specific fecundity (*m_x_*), and age-specific net maternity (*l_x_m_x_*) of *F. occidentalis* fed on Ca-treated leaves and control leaves are shown in Figure 2. The *l_x_* curve exhibited a decrease in survival with increasing thrips age. There were rapid declines in survivorship beginning at day 23 in the control group, whereas survivorship rapidly dropped beginning at day 20 in the Ca-treated group. The age-stage-specific fecundity curve (*f_xj_*) showed that female adults started reproducing at ages 12 d and 14 d when fed control and Ca-treated leaves, respectively. The peak of mean fecundity occurred on day 20, with 11.90 offspring in the control group and day 21, and with 7.48 offspring in the Ca-treated group.

**Figure 3:**
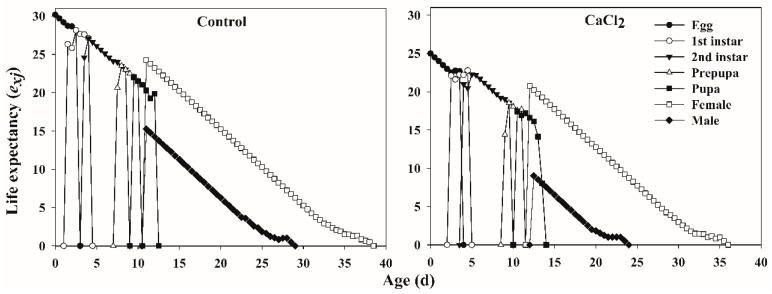
The age-stage-specific life expectancy (*e_xj_*) of *Frankliniella occidentalis* fed on control and Ca-treated kidney bean leaves.

**Figure 4:**
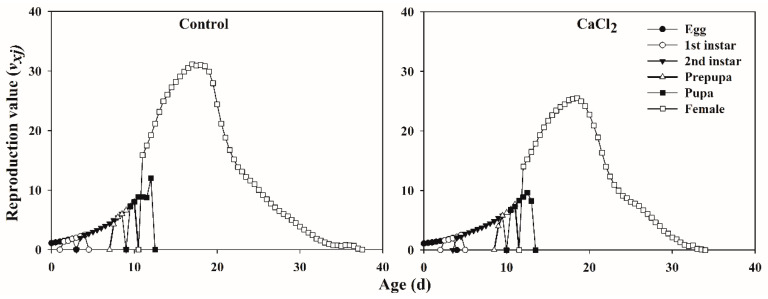
The age-stage-specific reproduction value (*v_xj_*) of *Frankliniella occidentalis* fed on control and Ca-treated kidney bean leaves.

The age-stage-specific life expectancies (*e_xj_*) of each age stage of *F. occidentalis* fed on Ca-treated leaves and control leaves gradually decreased with increasing thrips age (Figure 3). The life expectancies of egg, nymph, prepupa, pupa, and adult stages of *F. occidentalis* were higher when fed on control leaves than when fed on Ca-treated leaves. The age-stage-specific reproductive value (*v_xj_*) of *F. occidentalis* showed that the Ca treatment reduced the oviposition period and fecundity, compared with the control group (Figure 4).

#### 3.1.3. Population Parameters

The population parameters of *F. occidentalis* fed on Ca-treated leaves were significantly decreased compared with the population parameters of those fed on control leaves (Table 2). For example, the intrinsic rate of increase (*r*), finite rate of increase (*λ*), and net reproductive rate (*R*_0_) were all significantly higher in control leaves than in Ca-treated leaves, and the mean generation time (*T*) was significantly shorter (Table 2).

### 3.2. Choice Test

**Figure 5:**
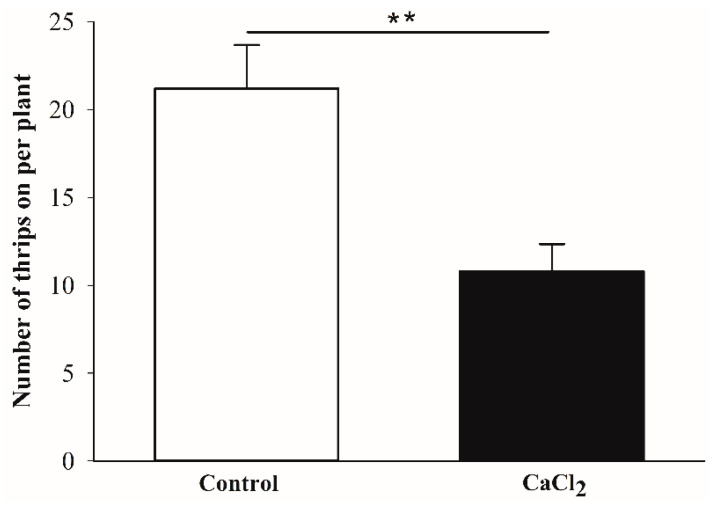
Number of adult female *Frankliniella occidentalis* found on control and exogenous Ca-treated kidney bean leaves after 24 h. Values are means ± SE. Asterisk indicate a significant difference (** p < 0.01).

The number of adult *F. occidentalis* on Ca-treated kidney bean plants was significantly lower than the number of adult *F. occidentalis* on control plants and was reduced by 49.1% (Figure 5; *χ*^2^ = 33.80, *df* = 1, N = 12, *p* < 0.01).

## 4. Discussion

*Frankliniella occidentalis* is a dominant and destructive pest worldwide in vegetable and flower production regions [2,3]. Various measures have been used to control thrips, but the use of pesticides is still the main method [3]. However, the use of pesticides leads to pest population resurgence, residue problems on crops, human health risks, toxicity to beneficial organisms, and environmental contamination [3,7]. Compared to the use of pesticides, sustainable management of thrips may involve the use of host plant defenses [9]. Plant’s induced defenses are rapidly gaining interest that may be exploited for the management of thrips [8,9]. Our previous study showed that kidney bean plants treated with Ca by spraying on leaves, root irrigation, or seed soaking could enhance defense enzyme activities in response to *F. occidentalis*, and the thrips’ growth period was also affected [26]. In another experiment, seed pretreatment with Ca upregulated expression levels of calmodulin (*CaM*) and callose synthase genes. The pretreatment extended aphid development time and reduced its feeding efficiency in wheat [25]. However, a full understanding of the Ca-induced plant defense will require the determination of the long-term performance of the herbivore. Here, we document the first report of a decrease in the longevity, fecundity, and other population parameters for *F. occidentalis* on kidney bean plants treated with exogenous Ca, as well as a reduced adult preference for these treated plants.

Numerous studies have demonstrated that exogenous biotic and abiotic elicitors have been used to induce host plant defense against *F. occidentalis*, for example, *Pseudomonas syringae*, coronatine [10], jasmonic acid (JA) [3,35], salicylic acid (SA) [36], ultraviolet [37], light intensity [38], nitrogen fertilizer [39], CO_2_ [40], secondary substances (α-ionone, eugenol, thymol, carvacrol and alkaloids, etc.) [41,42], and protein inhibitors [43]. In the present study, Ca treatment on kidney bean plants also influenced the population parameters and preference of *F. occidentalis*. To date, exogenous application of Ca has been reported to induce plant defenses against biotic stresses including bacterial wilt and powder mildew in tomato [22,23], leaf blast in wheat [21], Phytophthora stem rot in soybean [44], aphids in wheat [25], *E. saccharina* in sugarcane [24], and thrips in kidney bean [26].

Plants defend themselves against herbivores by employing chemical arsenals, including defensive enzymes and secondary metabolites [45]. Previous studies have shown that exogenous Ca plays an active role in plant resistance to biotic stress through upregulation of key defense enzyme activity [19]. Defense-related enzymes such as lipoxygenase (LOX), phenylalanine ammonia lyase (PAL), and β-1,3-glucanase are key enzymes involved in plant defense against insect herbivores [14,26,46]. In our previous study, the application of exogenous Ca enhanced the activity levels of the above enzymes in response to thrips attack [26]. PAL can induce plant cell wall lignification [11,47], while β-1,3-glucanase plays a crucial role in the remodeling of the plant cell wall [48], which might delay egg hatching from tissue, as observed in this study. LOX catalyzes the initial reaction in JA biosynthesis, which contributes to the biosynthesis of toxic secondary metabolites such as flavonoids, alkaloids, phenols, and glucosinolates [49,50,51,52]. These secondary metabolites might extend the development time of the immature stages and decrease the longevity and fecundity of adults when they are fed on Ca-pretreated kidney bean leaves by disrupting digestion and absorption or directly toxic action; however, this requires further investigation. In the immature stages, the second instar nymph is the main stage for intake of food [53]. It may be more affected than other stages due to intake of excess adverse substances, such as inducing low-dose secondary metabolites. The food consumption of the first instar nymphs is lower than the second instar nymphs, and the prepupa and pupa instars do not feed [53], resulting in less impact on growth parameters from Ca.

In general, the preference choice of herbivores for host plants has been useful to assess plant resistance [45,54]. For example, silencing the *CaLOX2* and *coi1-1* genes, which are involved in the defense of peppers and *Arabidopsis* against herbivores, enhanced the preference choice of *F. occidentalis*, compared to wild-type plants [3,35]. Exogenous application of potassium (K) to soybean significantly reduced the preference choice of soybean aphids (Hemiptera: Aphididae), compared to the control plants [55]. In rice, exogenous zinc (Zn) pretreatment affected adult white-backed planthopper (Hemiptera: Delphacidae) alightment [56]. Likewise, *F. occidentalis* showed a significantly lower preference for kidney bean plants treated with JA and carvacrol or thymol than for untreated plants [3,41]. In the present study, the putative induction of plant defenses by the application of exogenous Ca may have affected the preference choice of thrips, compared with the control plant. Such effects may alter the population distribution of *F. occidentalis* in crops by inducing a nonpreference mechanism, which may protect the crops from herbivores. 

In conclusion, our study demonstrates the negative effects of Ca-treated plants on *F. occidentalis*. Exogenous Ca on leaves of kidney bean plant could reduce the contemporary generation of *F. occidentalis* and its population quantity by decreasing its longevity, fecundity, and other population parameters. Longer-term impacts were not evaluated and require further study. Additionally, the impact of Ca-treated plants on *F. occidentalis* in the field requires investigation.

## Figures and Tables

**Table 1:** Developmental duration, survival, and reproduction of *Frankliniella occidentalis* fed on control and Ca-treated kidney bean leaves.

Parameter	n	Control	n	Ca-Treated
Egg (d)	60	2.55 ± 0.05 b	60	3.14 ± 0.04 a
First instar (d)	59	1.57 ± 0.03 a	57	1.58 ± 0.03 a
Second instar (d)	58	4.17 ± 0.05 b	54	4.79 ± 0.05 a
Prepupa (d)	57	1.51 ± 0.03 a	52	1.53 ± 0.04 a
Pupa (d)	56	1.75 ± 0.04 a	50	1.79 ± 0.05 a
Immature stages (d)	56	11.61 ± 0.06 b	50	12.82 ± 0.05 a
Preadult survival rate	56	0.93 ± 0.03 a	50	0.83 ± 0.05 a
Longevity/Female (d)	35	24.72 ± 0.26 a	32	19.94 ± 0.25 b
Longevity/Male (d)	21	14.52 ± 0.33 a	18	8.67 ± 0.28 b
APOP (d)	56	1.97 ± 0.09 a	50	2.0 ± 0. 0.13 a
TPOP (d)	56	13.51 ± 0.13 b	50	14.79 ± 0.14 a
Fecundity (eggs/female)	35	80.29 ± 7.29 a	32	55.34 ± 8.09 b
Oviposition days (d)	35	19.88 ± 0.14 a	32	16.14 ± 0.13 b

Values are mean ± SE. Means in a row followed by the different letters indicate significant differences between control and Ca-treated leaves based on the paired bootstrap test at the 5% level. APOP: the period between adult emergence and first oviposition; TPOP: the period from egg to adult plus preoviposition period.

**Table 2:** The population parameters of *Frankliniella occidentalis* fed on control and Ca-treated kidney bean leaves.

Treatments	*r* (d^−1^)	*λ* (d^−1^)	*R*_0_ (Offspring)	*T* (d^−1^)
Control	0.19 ± 0.01 a	1.21 ± 0.01 a	47.17 ± 5.20 a	19.93 ± 0.15 b
Ca-treated	0.16 ± 0.01 b	1.17 ± 0.01 b	29.62 ± 3.66 b	21.04 ± 0.13 a

Values are means ± SE. Means in a column followed by the different letters indicate significant differences between control and Ca-treated leaves based on the paired bootstrap test at the 5% level. *r*: intrinsic rate of increase; λ: finite rate of increase; *R*_0_: net reproductive rate; *T*: mean generation time.

## Data Availability

Data is available on request.
